# Synergistic pairing of synthetic image generation with disease classification modeling permits rapid digital classification tool development

**DOI:** 10.1038/s41598-024-77565-6

**Published:** 2024-10-27

**Authors:** Lao-Tzu Allan-Blitz, Sithira Ambepitiya, Janitha Prathapa, Cornelis A. Rietmeijer, Yudara Kularathne, Jeffrey D. Klausner

**Affiliations:** 1 Division of Global Health Equity, Department of Medicine, Brigham and Women’s Hospital, Boston, MA USA; 2 HeHealth Inc. San Francisco, CA San Francisco, USA; 3 Rietmeijer Consulting, Denver, CO USA; 4 Department of Population and Public Health Sciences, Keck School of Medicine, University of Southern California, Los Angeles, CA 90033 USA; 5 Department of Medicine, Harvard Medical School, Boston, MA 02115 USA

**Keywords:** Denoising diffusion probabilistic modeling, Text-to-image modeling, Machine-learning disease classification, Human papilloma virus, Artificial intelligence, Infectious diseases, Skin manifestations, Diagnosis

## Abstract

Machine-learning disease classification models have the potential to support diagnosis of various diseases. Pairing classification models with synthetic image generation may overcome barriers to developing classification models and permit their use in numerous contexts. Using 10 images of penises with human papilloma virus (HPV)-related disease, we trained a denoising diffusion probabilistic model. Combined with text-to-image generation, we produced 630 synthetic images, of which 500 were deemed plausible by expert clinicians. We used those images to train a Vision Transformer model. We assessed the model’s performance on clinical images of HPV-related disease (n = 70), diseases other than HPV (n = 70), and non-diseased images (n = 70), calculating recall, precision, F1-score, and Area Under the Receiver Operating Characteristics Curve (AUC). The model correctly classified 64 of 70 images of HPV-related disease, with a recall of 91.4% (95% CI 82.3%-96.8%). The precision of the model for HPV-related disease was 95.5% (95% CI 87.5%-99.1%), and the F1-score was 93.4%. The AUC for HPV-related disease was 0.99 (95% CI 0.98-1.0). Overall, the HPV-related disease classification model demonstrated excellent performance on clinical images, which was trained exclusively using synthetic images.

**Supplementary Information:**

The online version contains supplementary material available at 10.1038/s41598-024-77565-6.

## Introduction

Machine-learning models for disease classification have the potential to support the diagnosis of various diseases. Such models have been used to support radiographic assessment of different pathologies^1^, data-monitoring to classify patients with type 1 diabetes^2^, and clinical identification of cutaneous diseases^3^. However, the algorithms are dependent on large clinical image databases encompassing a diverse range of pathology, which are not available for many diseases, and can be difficult to access due to patient privacy protections. Further, the establishment of such databases is time- and resource-intensive, limiting the potential applications of machine-learning diagnostic models. To offset the need for large datasets of clinical images for any given disease, some have explored synthetic image generation from non-image inputs using machine-learning models – the converse of image classification. The potential applications of such a synergistic pairing of image generation and image classification models are numerous. During the outbreak of a new cutaneous disease, similar to the 2022 mpox outbreak^4^, when timely diagnosis is essential to limit the spread of infection, dependency on clinical image databases to support machine-learning models may limit utility.

The traditional method for image generation is known as generative adversarial networks (GAN)^5^. GANs have numerous limitations, however, including a lack of image diversity and dependence on large datasets, which have limited their value in medical contexts^6,7^. A novel approach to image generation, known as diffusion probabilistic modeling, has had much more success than GANs^8,9^, which, with additional improvements, can reverse the diffusion process, effectively transforming image ‘noise’, or uninterpretable pixels, into coherent imagery^10^. Recent advances have led to improved denoising of diffusion probabilistic models^11^, further augmenting the quality of synthetic image generation. One study applied denoising diffusion probabilistic modeling to a dataset of magnetic resonance and computed tomography images, and demonstrated robust, diverse, and high-quality production of 3-dimensional images, as evaluated by expert radiologists^12^. That study then used the images generated to train an existing breast image segmentation model, resulting in improved model performance^12^.

We aimed to further enhance denoising diffusion probabilistic modeling, adding text-to-image generation and train a Vision Transformer model for disease classification using entirely synthetic, generated images. We selected genital warts, caused by human papilloma virus (HPV) infection, as a use case. HPV-related disease is highly stigmatized^13^ for which visual diagnosis is a core component. Further, HPV-related diseases are common, with an estimated 10% lifetime cumulative incidence^14^. A disease classification model for genital warts has the potential to support the diagnosis of this highly prevalent and morbid disease.

## Methods

### Synthetic image generation

We curated an clinical image dataset of 10 penises with HPV-related disease sourced from a mobile platform we previously described^15^. Two expert clinicians reviewed each image to confirm representation of HPV-related disease. We paired those images with descriptive text files to link subject class (e.g., genital warts) with text prompt descriptors (e.g., “cauliflower shape”). We then applied class-specific prior-preservation loss to encourage generation of diverse images per class, as has been described previously^16^. We also used a bounding box to shift the generative model’s focus to the HPV-related lesions, rather than the entire genital area, as we found the broader focus resulted in inaccurate rendering of the genital area during model piloting.

We inputted those synthetic images into a diffusion probabilistic model using stable diffusion to create coherent structured images from random image noise. We further enhanced that model with an existing personalized text-to-image model known as DreamBooth^17^, which permitted generation of customizable synthetic content based on text descriptions. To do so, we used a U-net architecture model to classify pixels as either background or subject image^18^, as well as a text encoder training as a part of the DreamBooth software. The U-net learning rate (or model change in response to estimated error each time the weights are updated) was fixed at 2e^−6^, while the text encoder learning rate was set at 4e^−7^ with training steps set at 350 to balance learning with overfitting.

**Fig. 1:**
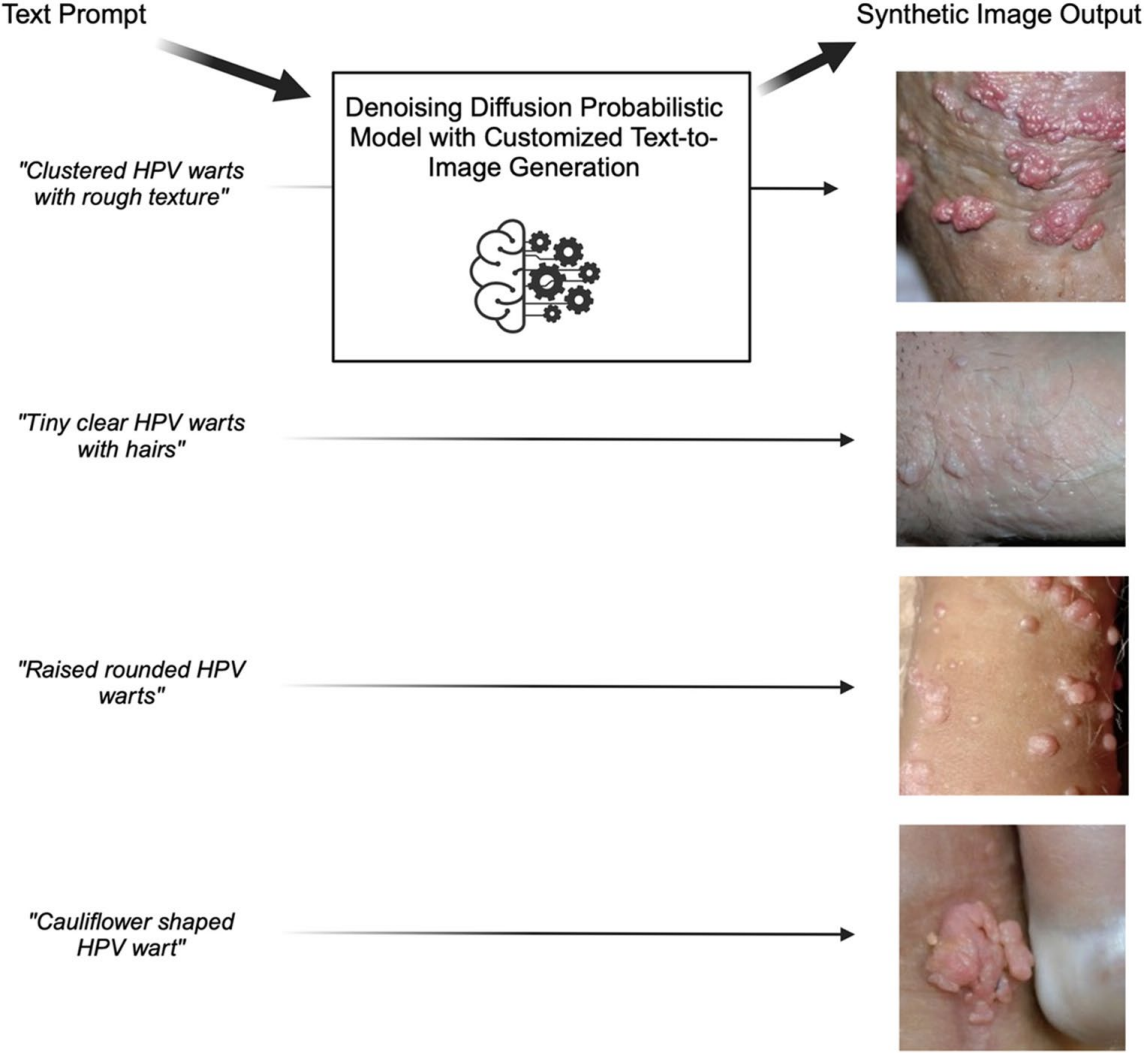
Examples of input text and output images from text-to-image generation.

We then used text to specify a range of HPV-related disease presentations as well as multiple variations on the same pathology to generate images representing the complete disease spectrum. The Fig. 1 shows examples of text inputs and image output.

To determine clinical plausibility, expert clinicians assessed each image. All images were reviewed first by one physician (a general practitioner with expertise in dermatologic conditions). The physician categorized each image as either clinically plausible or not. A further random sample of 20 images (10 categorized as clinically plausible and 10 categorized as clinically implausible) were sent to a second clinician, a professor of dermatology, for a blinded second review. We used the resulting synthetic images deemed plausible to train the classification model. Supplemental Fig. 1 in the Appendix shows examples of images deemed implausible.

### Disease classification model

We used a Vision Transformer model for learning on the synthetic images^19^. Such models deconstruct images into segments, and analyze each segment individually to make a final determination about whether or not the disease is present. We used the Vision Transformer Base-Patch 16–224 (Google Brain, United States), which processes images in 16 × 16 pixel patches and is optimized for an input size of 224 × 224 pixels. We used the cross-entropy loss function to train the classifier; the cross-entropy loss function measures model performance by comparing the predicted class probability with the actual class labels, and uses the output to guide optimization. No further image segmentation training was required as all synthetic images were produced with labels of disease characteristics, on which the Vision Transformer model relied.

We evaluated the addition of two commonly used image optimizers to reduce information losses, which function via augmentation of neural network attributes (e.g., weights, learning rate, etc.): Adam optimizer (Adaptive Moment Estimation)^20^, and Root Mean Squared Propagation image optimizer^21^. We set the epochs at 50 for that comparison. Subsequently, we serially evaluated model epochs (number of times the model works through the entire training set) between 50 and 200 at intervals of 50. After determining the optimal epochs, we evaluated model learning rates between 1e^−2^ and 1e^−5^. We used the average F1-score (see below) as the outcome metric for each of those comparisons^22^. For all model optimizations, we used a subset of images from the training dataset.

We trained that model exclusively on the 500 synthetic images of HPV-related disease, as well as 500 images of non-diseased penises and 500 images of other penile pathology (herpes simplex virus (HSV) (*n* = 150), primary syphilis (*n* = 150), penile cancer (*n* = 140), penile eczema (*n* = 30), and penile psoriasis (*n* = 30)). All clinical images were submitted by clinicians, including experts in infectious diseases, sexually transmitted diseases, dermatology, or family practice with a special interest in sexually transmitted diseases from six countries (India, Sri Lanka, Singapore, Australia, The United States, and The United Kingdom).

**Fig. 2:**
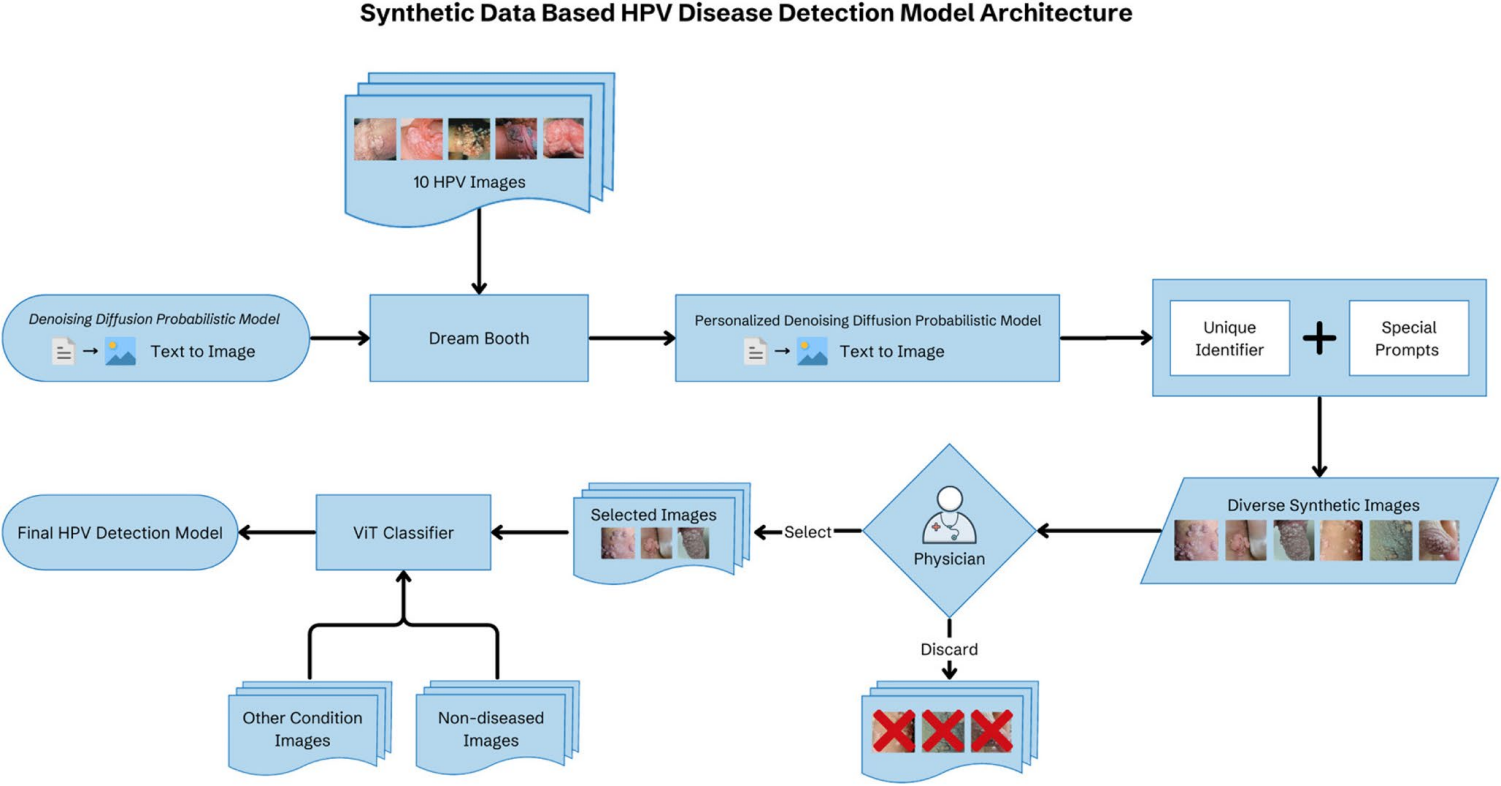
Schematic showing model architecture pairing text-to-image inputs with denoising diffusion probabilistic modeling to generate synthetic images of HPV-related disease used to train a vision transformer classification model. Legend for Fig. 2: The figure shows the overall structure of the HPV-related disease classification model. Text-to-image inputs paired with a denoising diffusion probabilistic model generated synthetic images across a range of presentations. All generated images were then reviewed by an expert physician, and improbable synthetic images were removed. The remaining synthetic images were used as inputs in a vision transformer classification model along with clinical images of other similar diseases and non-diseased images.

For further fine tuning of the model, we conducted a validation step using 50 images previously not seen by the model in each class (HPV-related disease, non-HPV-related disease, and non-diseased images). The 50 images of other pathology included 20 of HSV infection, 10 of primary syphilis, 10 of penile cancer, 5 of penile eczema, and 5 of penile psoriasis. We took several steps to guard against overfitting during training. First, we aimed to ensure a diverse sample set using text-to-image prompts. Second, we employed early stopping, whereby training stops when loss ceases to improve in the validation dataset after a set number of epochs. Finally, we maintained a separate validation dataset. Figure 2 shows the overall model architecture.

### Assessment of model performance

We assessed the performance of the model on an additional dataset of clinical images. We used 70 clinical images of HPV-diseased penises, 70 of diseases other than HPV (HSV (*n* = 30), primary syphilis (n-20), penile cancer (*n* = 10), penile eczema (*n* = 5), and penile psoriasis (*n* = 5)), and 70 non-diseased images. All images were submitted by expert clinicians. We then assessed the performance of the classification model against all 210 images, calculating recall (or sensitivity), precision (or positive predictive value), specificity, and F1-score^22^, as well as overall accuracy of the model, which we defined as the average F1-score across each disease class. Equations for each outcome metric are shown below.

We graphed the performance of the model via a Receiver Operating Characteristic curve using TensorFlow Version 2.8.0 (TensorFlow, United States). We used TensorFlow to calculate the Area Under the Curve (AUC) for multiclass classifications, which compared the performance of the model for each class (e.g., HPV-related disease images) against the combination of all other classes (i.e., HPV-unrelated disease images combined with non-diseased images). We therefore calculated the AUC of the model for each classification. As a sensitivity analysis, we developed additional validation datasets via bootstrap resampling of the initial dataset over 1,000 iterations, and recalculated the AUC for each classification. We report the average AUC and 95% confidence intervals (CI) across all 1,000 iterations.

### Ethical considerations

All methods were carried out in accordance with the relevant guidelines and regulations, which included the reporting guidelines for prognostic and diagnostic machine learning modeling studies^23^. Informed consent was obtained from all subjects who submitted clinical images through the mobile platform prior to use. All experimental protocols for this study were approved by the National University of Singapore institutional review board. The analysis of de-identified data was deemed non-human subjects’ research by the Massachusetts General Brigham institutional review board.

### Equations

$$\:Recall\:\left(Sensitivity\right)=\frac{True\:Positive}{(True\:Positive+False\:Negative)}$$

$$\:Precision\:\left(Positive\:Predictive\:Value\right)=\frac{True\:Positive}{(True\:Positive+False\:Positive)}$$

$$\:F1\:Score=2*\frac{\left(Precision*Recall\right)}{\left(Precision+Recall\right)}$$

## Results

The denoising diffusion probabilistic model produced 630 synthetic images. Of those, 500 were deemed plausible by the first expert reviewer. A blinded, second expert reviewer agreed with the plausibility determination of all 20 randomly selected images (10 plausible, 10 implausible).

Table 1 shows the results of model optimization. Between the two image optimizers, we selected Root Mean Squared Propagation image optimizer given trend towards improved accuracy. Using that optimizer, epochs of 150 appeared to perform optimally, with a learning rate of 1e^−4^. Supplemental Fig. 2 in the Appendix shows the learning curves for both the training and validation datasets, which indicate successful mitigation of overfitting.

**Table 1:** Model optimization via selection of image optimizer, epochs, and learning rate.

No. Epochs	Optimizer	Learning Rate	No. (%) HPV-Related Disease Images Correctly Classified (*n* = 70)	No. (%) Other Disease Images Correctly Classified (*n* = 70)	No. (%) Non-Diseased Images Correctly Classified (*n* = 70)	Average F1 Score (95% CI)
50	Adam	1.00E-03	61	62	66	90.0% (85.1 − 93.7%)
50	RMSprop	1.00E-03	63	61	67	91.0% (86.2 − 94.5%)
50	RMSprop	1.00E-03	63	61	67	91.0% (86.2 − 94.5%)
100	RMSprop	1.00E-03	63	62	68	91.9% (87.4 − 95.2%)
150	RMSprop	1.00E-03	63	63	69	93.0% (88.5 − 96.0%)
200	RMSprop	1.00E-03	63	61	69	91.9% (87.4 − 95.2%)
150	RMSprop	1.00E-03	63	63	69	93.0% (88.5 − 96.0%)
150	RMSprop	1.00E-04	64	64	70	94.3% (90.2 − 97.0%)
150	RMSprop	1.00E-05	62	61	68	91.0% (86.2 − 94.5%)

**Fig. 3:**
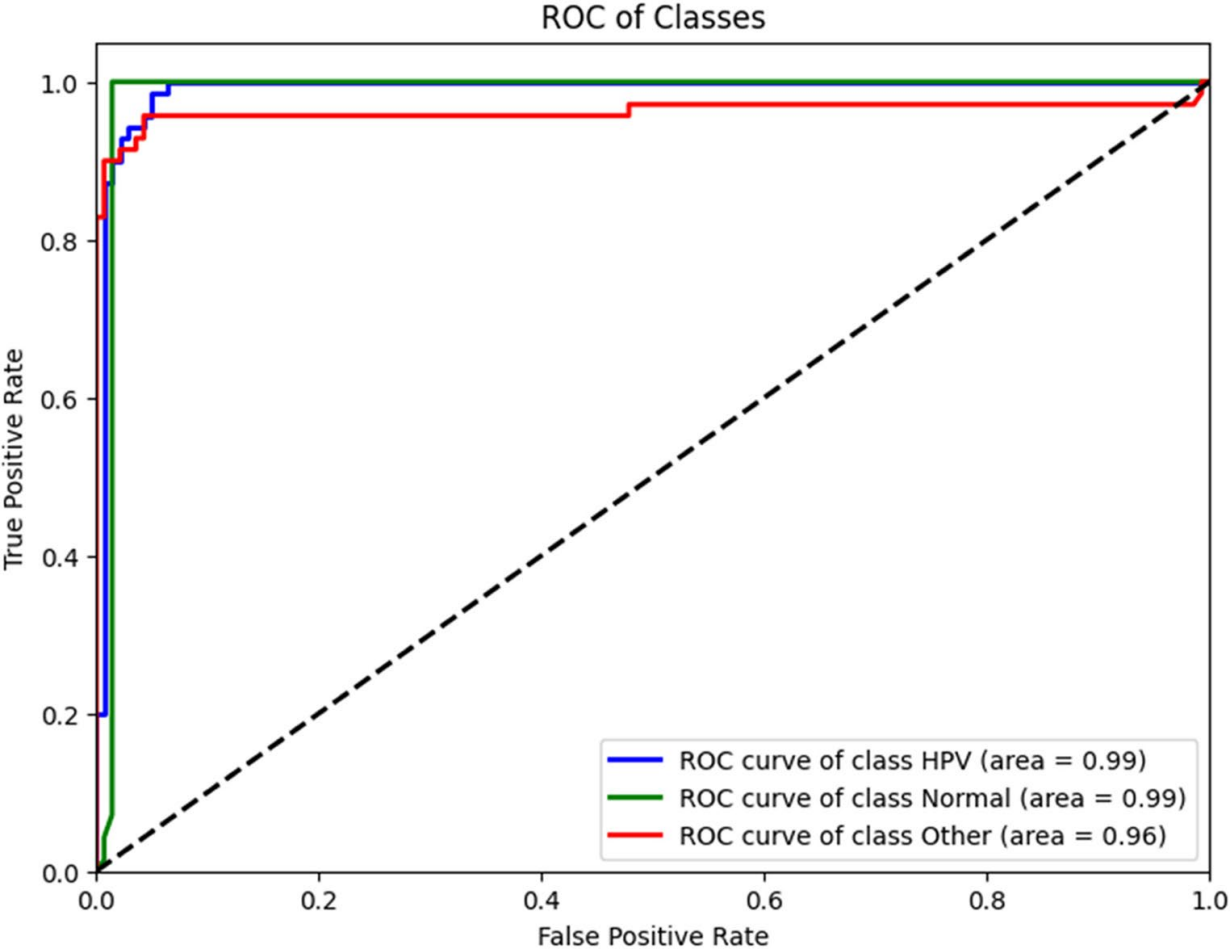
Receiver operating characteristics curve for HPV-related disease classification model trained exclusively on synthetic images. Legend for Figure 3: The figure shows the receiver operating characteristics curve for the machine-learning HPV-related disease classification model, plotting model sensitivity against 1 minus the specificity.

The model correctly classified 64 of 70 HPV-related disease images, with a recall (or sensitivity) of 91.4% (94% CI 82.3% – 96.8%). The precision (or positive predictive value) of the model for HPV-related disease was 95.5% (95% CI 87.5% − 99.1%), and the F1-score was 93.4%. Table 2 shows the distribution of images and model assignment. Figure 3 shows the Receiver Operating Characteristics curve of the model performance for each class; the AUC of the model for images of HPV-related disease was 0.99 (95% CI 0.98-1.0%), with similar AUCs for images of HPV-unrelated disease (0.96; 95% CI 0.93–0.99) and non-diseased images (AUC 0.99; 95% CI 0.98-1.0). The performance of the model was stable across all 1,000 bootstrap iterations for all three classifications: average HPV-related disease AUC 0.99 (95% CI 0.98-1.0), average HPV-unrelated disease AUC 0.96 (95% CI 0.91–0.99), and average non-diseased AUC 0.99 (95% CI 0.96-1.0).

**Table 2:** Performance of machine-learning model for classifying Penile HPV-related disease trained exclusively on synthetically-generated images.

	HPV-Related Disease Images	Other Pathology^†^	Non-Diseased Images
Model Classified as HPV-Related Disease Image	64	3	0
Model Classified as Other Pathology	5	64	0
Model Classified as Non-Diseased Image	1	3	70
Total	70	70	70

† Non-HPV-related pathology images included herpes eruption (*n* = 30), primary syphilis (*n* = 20), penile cancer (*n* = 10), penile eczema (*n* = 5), and penile psoriasis (*n* = 5).

**Fig. 4:**
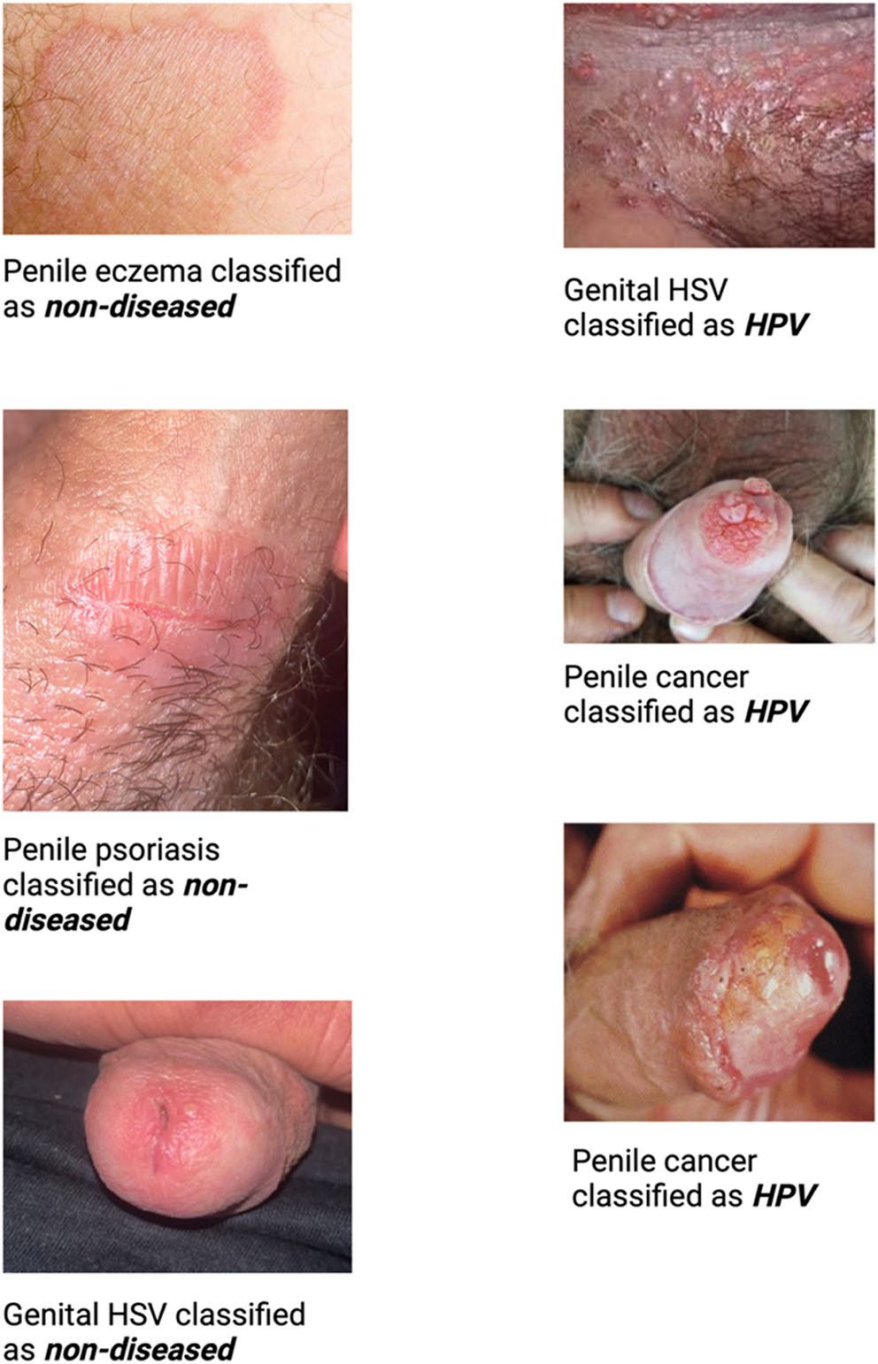
Images incorrectly classified by the machine-learning model. Legend for Fig. 4: The figure shows six images misclassified by the machine-learning HPV classification model, one image of genital herpes incorrectly classified as HPV-related disease, two images of penile cancer incorrectly classified as HPV-related disease, and one image of penile eczema, penile psoriasis, and genital HSV incorrectly classified as non-diseased.

The model incorrectly classified six images of genital warts, one as non-diseased and five as other non-HPV-related pathology. The model also incorrectly identified one image of HSV infection as HPV-related disease, and two images of penile cancer as HPV-related disease, as well as one image of HSV infection, one image of penile psoriasis, and one image of penile eczema as non-diseased. Figure 4 shows the misclassified images. Supplemental Fig. 3 in the Appendix shows additional examples of images correctly classified.

## Discussion

We developed a machine-learning disease classification model for external penile warts that was trained exclusively on synthetically generated images using a combination of denoising diffusion probabilistic and of text-to-image models. That model demonstrated excellent performance against a validation dataset of a range of clinical images. The described approach pairing synthetic image generation and disease classification has numerous possible extensions, including improved support for visually classifying known diseases as well as quickly developing classification models for new and emerging diseases. Additionally, with further development and evaluation, the specific model produced has the potential to support HPV-related disease diagnosis, particularly in areas with limited access to specialists.

Recent work has explored similar pairing of synthetic image generation and downstream classification. *Schaudt* et al. evaluated five different image generation models for abnormal chest radiograph images and assessed performance on a subsequent classification task, reporting accuracies ranging from 73 to 81%^24^. Notably, that study used intentionally simple text inputs to isolate the impact of image augmentation after generation, which may have impacted overall performance of the classification model^24^. Another study used a GAN model to produce synthetic images of retinal blood vessels and successfully trained classification models for retinal pathology, which showed an AUC above 97%^25^. However, that dataset required training on nearly 3,500 clinical images. Using denoising diffusion probabilistic modeling with more elaborate text inputs permitted training with only 10 clinical images, and resulted in an AUC above 93%.

Our HPV-related disease classification model has many possible applications. Incorporation of the model into a mobile platform can facilitate at-home disease classification, possibly overcoming fear of stigma that restrict healthcare seeking behavior^26,27^. Use in low-resource settings can support access to specialized sexual health services^27^. Given the inherent connectivity of mobile platform-based diagnoses, such tools can also support case identification and public health surveillance.

Potential impacts of the overall approach – combining synthetic image generation to rapidly train a classification model on a limited initial dataset – are broad. In the context of an outbreak of a new or emerging disease, initial image repositories are often non-existent. Creation of such datasets is labor and time-intensive, limiting any possible utility of machine-learning models to support diagnosis. Our approach overcomes that barrier, permitting the development and deployment of models with minimal initial data input.

Medical image repositories are also difficult to establish because of patient privacy protections. Synthetic images are not subject to the same restrictions. Other areas of medicine that can benefit from such an approach include the incorporation of machine-learning to support radiographic evaluation of two-dimensional images (e.g., chest radiographs) or 3-dimensional images (e.g., computed tomography and magnet resonance images)^1^, and even clinical dentistry^28^. Thus, the current successful pairing of synthetic image generation and disease classification represents a significant advance, opening the door for many other applications of machine-learning models into healthcare.

## Limitations

Our study had several limitations. First, the model was assessed on a relatively small sample of clinical images, limiting the precision of our findings. Second, while the model maintained classification integrity against several distinct disease states, there remain additional pathologies that can mimic genital warts (e.g., molluscum contagiosum, condyloma lata of secondary syphilis, etc.), and thus potentially attenuate model performance. Subsequent work should aim to refine the model via including additional diseases in model training, evaluating the differences in model performance by skin tone, image quality, and type of image used, and aim to refine the synthetic image generation in order to reduce the number of implausible images produced. Reducing the number of implausible images produced will be additionally helpful for improving the usefulness of the approach described. As we did not have data on the reasons for classifying an image as implausible, such should be the subject of future work.

Further, the performance of the model was assessed on submitted clinical images with a balanced distribution of diseases. Additional methods of evaluating model accuracy will be important to understand model performance and possible utility; the model could be compared, for example, against expert physician classification of such images, blinded to the diagnosis. Additionally, the model could be assessed against molecular diagnostic testing (e.g., polymerase chain reaction of lesion swabs). Future work could also develop alternative image generation methods for comparison in order to identify the optimal strategy. Real-world evaluation will support evaluation among populations with varying disease prevalence, as well as further evaluate if the range of HPV-related disease images sufficiently captured the diversity clinical presentations. Therefore, further evaluation in a real-world setting is warranted. However, given the potential impact of pairing image generation with classification model training, however, we feel those limitations do not diminish the importance of our findings.

## Conclusion

We paired denoising diffusion probabilistic modeling with a text-to-image model to produce synthetic images based on minimal initial training, which we used to train a downstream disease classification model without any further augmentation. That model demonstrated excellent performance for classifying images of penile pathology. The potential applications of this successful synergistic pairing are numerous.

## Electronic supplementary material

Below is the link to the electronic supplementary material.

Supplementary Material 1

## Data Availability

All data supporting this manuscript are available upon reasonable request of the corresponding authors.
