# Supplementary material for: Synergistic pairing of synthetic image generation with disease classification modeling permits rapid digital classification tool development
Source: Sci Rep. 2024 Oct 27;14:25632. doi: 10.1038/s41598-024-77565-6 (PMC11514197; doi:10.1038/s41598-024-77565-6)
Supplement: Supplementary file 1 — Supplementary Material 1 [file 41598_2024_77565_MOESM1_ESM.docx]

**Appendix**

**
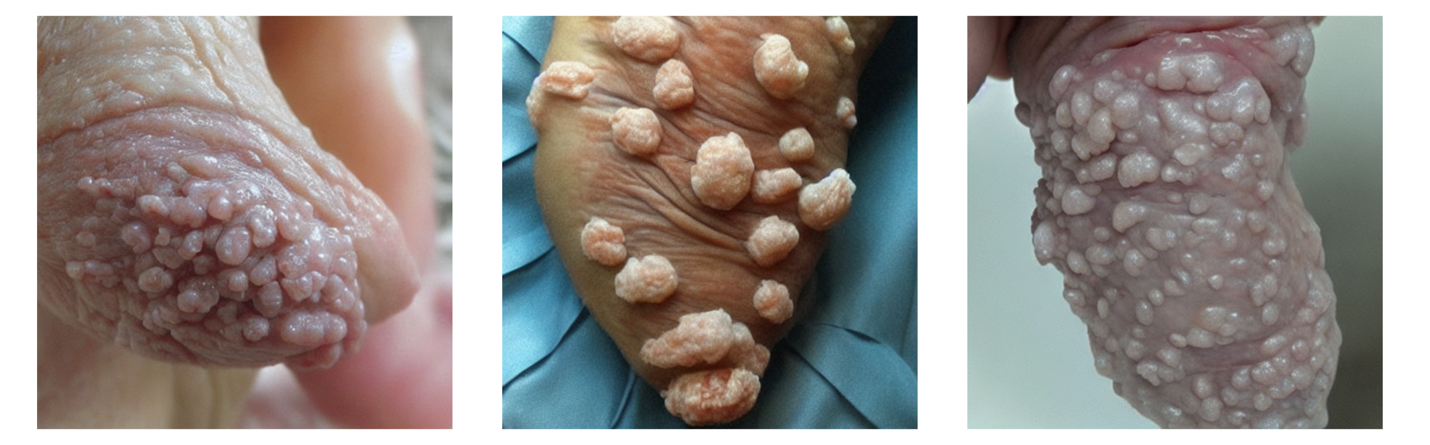
Supplemental Figure 1: Example Synthetic Images Deemed Implausible by Expert Clinician Review**

**Supplemental Figure 2: Learning Curves Showing Loss in Training and Validation Datasets**

**Panel A**

**
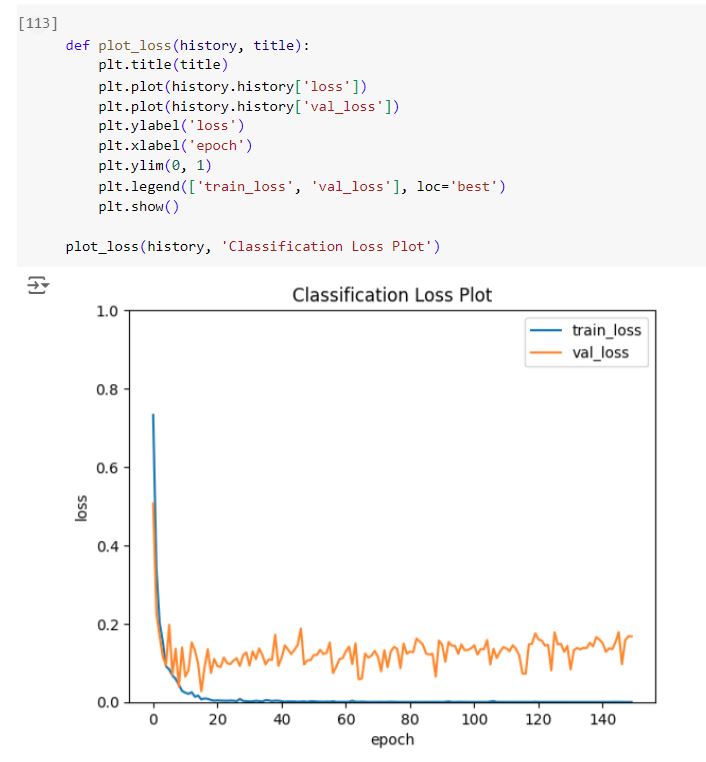
**

**Panel B**


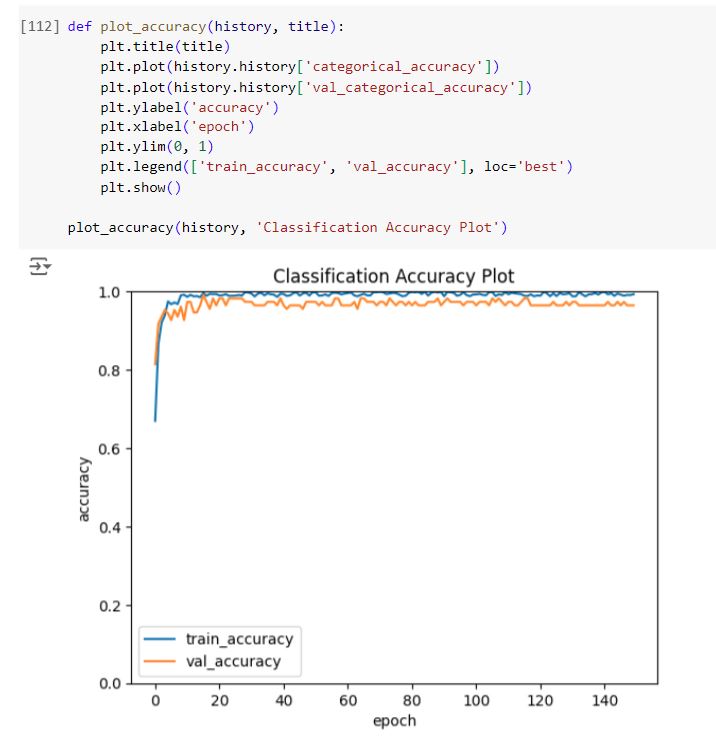


Legend for Supplemental Figure 2: Panel A shows the change in loss by epochs for the training dataset (blue) and the validation dataset (orange). Panel B shows the change in accuracy by epochs for both datasets.

**Supplemental Figure 3: Examples of Images Correctly Classified as HPV-related disease by the Machine-Learning Model**

**
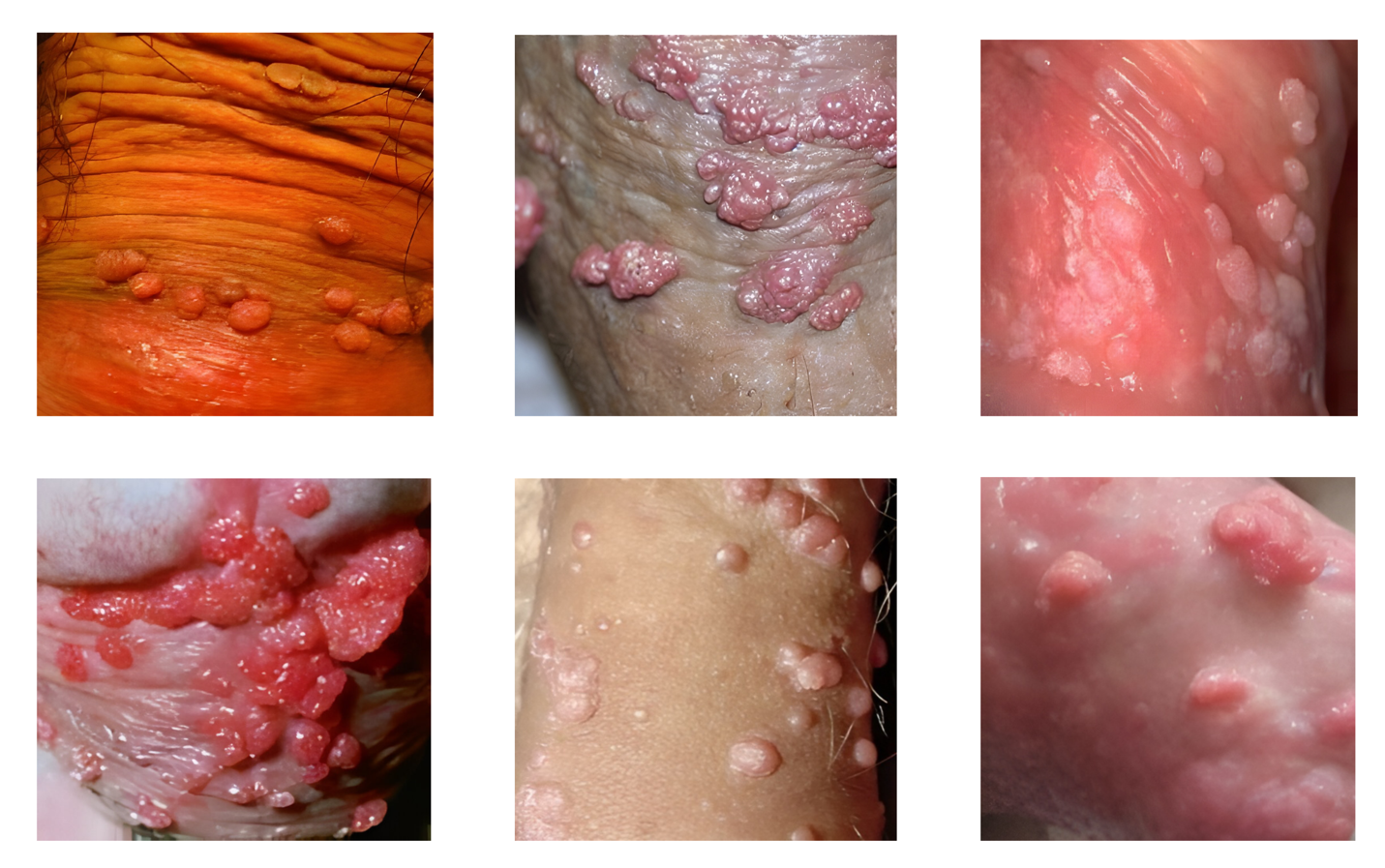
**

**Glossary of Terms**

*In order of appearance*

**Class-specific prior-preservation loss** – Class-specific, or in this case disease specific (e.g., HPV-related disease vs non-HPV disease vs non-diseased) ‘loss’ is a measure of how distinct one generated image is from another. Prior-preservation loss is a technique to ensure the model learns to minimize loss in order to generate diverse images.

**Diffusion probabilistic model** – A machine-learning technique that evolves random noise into structured, coherent images or patterns. This gradually shapes unstructured data into detailed visuals, making it highly effective for generating realistic images and intricate data visualizations.

**DreamBooth** – An existing technique for personalized text-to-image generation.

**U-net architecture model** – A type of neural network architecture commonly used in image processing tasks that classifies pixels as either background or subject image.

**Learning rate** – A parameter of machine-learning models that determines how much the model changes the weights of the U-net architecture in response to estimated error.

**Training steps** – The number of times the model parameters are permitted to be updated.

**Vision Transformer model** – A machine-learning classification model that deconstructs images into segments, analyzing each segment individually, and ultimately making a final determination of the image classification based on the predetermined classes.

**Image optimizers** – Algorithms designed to minimize the loss function during training of neural networks. Examples include the Adam (Adaptive Moment Estimation) and Root Mean Squared Propagation image optimizers.

**Epochs** – The number of times the model works through the entire training dataset.
